# A Decline in the Incidence of Invasive Non-Typhoidal *Salmonella* Infection in the Gambia Temporally Associated with a Decline in Malaria Infection

**DOI:** 10.1371/journal.pone.0010568

**Published:** 2010-05-11

**Authors:** Grant Mackenzie, Serign J. Ceesay, Philip C. Hill, Michael Walther, Kalifa A. Bojang, Judith Satoguina, Godwin Enwere, Umberto D'Alessandro, Debasish Saha, Usman N. A. Ikumapayi, Tim O'Dempsey, David C. W. Mabey, Tumani Corrah, David J. Conway, Richard A. Adegbola, Brian M. Greenwood

**Affiliations:** 1 Medical Research Council (United Kingdom) The Gambia, Fajara, The Gambia; 2 Centre for International Health, University of Otago, Dunedin, New Zealand; 3 Meningitis Vaccine Program, Program for Appropriate Technology in Health, Ferney-Voltaire, France; 4 Institute of Tropical Medicine, Antwerp, Belgium; 5 Liverpool School of Tropical Medicine, Liverpool, United Kingdom; 6 London School of Hygiene and Tropical Medicine, London, United Kingdom; 7 Bill & Melinda Gates Foundation, Seattle, Washington, United States of America; The George Washington University Medical Center, United States of America

## Abstract

**Background:**

Malaria is a risk factor for invasive non-typhoidal *Salmonella* (NTS) infection in children. In the last 10 years, indices of malaria infection in The Gambia have fallen substantially.

**Methods:**

We compared temporal trends of childhood malaria and NTS infection in two Gambian locations. In Fajara, on the coast, the incidence of NTS infection at three time points between 1979 and 2005 was compared to the percentage of malaria positive outpatient thick blood films and the percentage of admissions associated with malaria over time. In Basse, in the eastern part of the country, the incidence of NTS infection at three time points between 1989 and 2008 was compared to the prevalence of malaria parasitaemia at four time points between 1992 and 2008.

**Results:**

The estimated incidence of NTS infection in Fajara fell from 60 (1979–1984) to 10 (2003–05) cases per 100,000 person years. The proportion of outpatients in Fajara with suspected malaria who were parasitaemic fell from 33% (1999) to 6% (2007) while the proportion of admissions associated with malaria fell from 14.5% (1999) to 5% (2007). In Basse, the estimated incidence of NTS infection fell from 105 (1989–1991) to 29 (2008) cases per 100,000 person years while the prevalence of malaria parasitaemia fell from 45% (1992) to 10% (2008). The incidence of pneumococcal bacteraemia in Fajara and Basse did not fall over the study period.

**Conclusions:**

These data support an association between malaria and NTS infection. Reductions in malaria infection may be associated with reduced rates of invasive childhood NTS infection.

## Introduction

Non-typhoidal *Salmonella* species (NTS) are a leading cause of invasive bacterial infection in African children and are responsible for many deaths [1], [2]. Invasive NTS infection is difficult to manage, and is often complicated by resistance to commonly used antibiotics [3].

Evidence suggests that malaria is a risk factor for invasive NTS infection in children. In regions of seasonal malaria transmission, annual peaks of NTS and malaria infection coincide [4]–[7]. Invasive NTS is frequently associated with recent [5] or concurrent malaria infection [4], [7], [8], particularly severe malarial anemia [1], [4], [7], [9], [10].

Over the last 10 years, indices of malaria infection in The Gambia have fallen substantially [11]. If malaria is a risk factor for NTS infection, it would be anticipated that a fall in malaria incidence would be associated with reduced rates of NTS infection. To investigate this hypothesis we reviewed data on malaria and NTS infection in two regions of The Gambia collected during the past 30 years.

## Methods

We reviewed data from studies conducted in two locations in The Gambia, Fajara and Basse. The Gambian climate is sub-tropical with one wet season (June to November) during which most malaria transmission occurs. Almost all cases of malaria are due to *Plasmodium falciparum*. The Medical Research Council (MRC) Laboratories in Fajara, on the Atlantic coast, have provided clinical services for the surrounding population since the late 1950s. Basse is situated in the rural eastern part of the country about 400 km from the coast, where MRC has conducted studies since 1982.

To relate indices of invasive NTS and malaria infection in Fajara, four data sets were used (tables 1 and 2). These were (a) blood cultures taken for clinical purposes from all unwell children under 5 years of age admitted to the MRC hospital in Fajara between 1979 and 1984 [4], (b) blood cultures obtained from children aged 2–35 months with suspected meningitis, sepsis or pneumonia during a large-scale trial of *Haemophilus influenzae* type b (Hib) conjugate vaccine conducted in Western Region of The Gambia from 1991 till 1995 [12], (c) blood cultures collected for clinical purposes from all unwell patients aged less than 5 years admitted to the MRC hospital between 2003 and 2005 [13], and (d) routine malaria blood films obtained from patients who presented as outpatients at the MRC hospital with fever or other symptoms associated with malaria and also hospital admission records throughout the observation period [11].

**Table 1:** Characteristics of studies included in the analysis.

Ref.	Location	Year	Population	Design	Laboratory method	Outcome
[4]	Fajara	1979–84	Admissions <5 years of age	Observational	Conventional blood culture	Incidence NTS and IPD
[12]	Western	1993–95	Out/inpatients 2–35 months of age	RCT	Conventional blood culture	Incidence NTS and IPD
[13]	Fajara	2003–05	Admissions <5 years of age	Observational	Automated blood culture	Incidence NTS and IPD
*	Fajara	1982 & 1994	Outpatients	Observational	Positive malaria slide	Percentage slides positive
[11]	Fajara	1999–2007	Outpatients/inpatients	Observational	Positive malaria slide	Percentage slides positivePercentage malaria admissions
[6], [15]	URR	1989–91	Outpatients <5 years of age	Observational	Conventional blood culture	Incidence NTS and IPD
[14], [16]	U/CRR	2000–04	Out/inpatients 2–29 months of age	RCT	Automated blood culture	Incidence NTS and IPD
*	URR	2008	Out/inpatients <5 years of age	Observational	Automated blood culture	Incidence NTS and IPD
[17]	URR	1992	Community <5 years of age	Survey	Positive malaria slide	Prevalence malaria parasitaemia
*	URR	1994	Clinical trial 15–20 months of age	Survey	Positive malaria slide	Prevalence malaria parasitaemia
[18]	URR	1995	Clinical trial 27–32 months of age	Survey	Positive malaria slide	Prevalence malaria parasitaemia
[19]	URR	2008	Community <5 years of age	Survey	Positive malaria slide	Prevalence malaria parasitaemia

Note: * authors' unpublished data. URR, Upper River Region. CRR, Central River Region.

**Table 2:** Data derived from Fajara, Western Region studies.

Ref.	Location	Year	Population & (person-years) denominators	NTS isolates	Pneumococcal isolates	NTS incidence <5 years*	IPD incidence <5 years*	Percentage malaria slides positive	Percentage malaria admissions
[4]	Fajara	1979–84	20000 (115000)	69	38	60	33	n/a	n/a
[12]	Western	1993–95	(64181, 0–3 years)	39†	76‡	36§	70∥	n/a	n/a
[13]	Fajara	2003–05	38322 (51096)	5	29	10	57	n/a	n/a
†	Fajara	19821994	n/a	n/a	n/a	n/a	n/a	22%32%	
[11]	Fajara	19992001200320052007	n/a	n/a	n/a	n/a	n/a	33%32%22%8%6%	14.5%20.5%17%8.5%5%

Note: * Incidence per 100000 person-years.

†Authors' unpublished data (BG).

‡76/116 (66%) pneumococcal isolates were from blood cultures.

§As estimates of age-specific NTS infection are not available, the incidence of NTS disease is estimated from the ratio of NTS to pneumococcal isolates, 39:76 = 0.51, and the estimated incidence of pneumococcal blood stream infection.

∥Incidence of pneumococcal blood stream infection is calculated from values reported by Usen et al. 1998 [12] and the proportion of IPD due to blood stream infection (0.66), as 0–1 yr: 178×0.66 = 117, 2 yrs: 86×0.66 = 57, 3–4 yrs: extrapolated as half the incidence among those 2 yrs of age; 57/2 = 29, <5 yrs: [(117×2)+57+(29×2)]/5 = 70. § Incidence of NTS disease is calculated as 70×0.51 = 36.

Manual blood culture methods were used in the first two studies, after which an automated system was used. *Salmonella typhi* and non*-typhi* isolates were differentiated in the two hospital-based studies but not during the vaccine trial. However, *S. typhi* infections are very uncommon in young children in The Gambia [13], [14]. Thick blood films were read by experienced technicians and examination was undertaken on 100 high-power fields (1000 times magnification) for each slide throughout the observation period.

To determine the approximate incidence of blood culture positive NTS and invasive pneumococcal disease (IPD), an estimate was made of the catchment population for the MRC Hospital during the 1979-84 study (100000 with 20% aged less than 5 years). Population growth of 3% per year was used to give an estimated population for the 2003–2005 study. Because children in the Hib vaccine trial were aged 2 to 35 months, the overall incidence of IPD in children aged less than 5 years during the vaccine trial was estimated based on reported age-specific rates of IPD. Age-specific rates of IPD and NTS infection were extrapolated using data from two Kenyan studies which documented that the incidence of invasive IPD and NTS among children aged 36-59 months was approximately half the incidence among those aged 24–35 months [2], [5]. The population aged 3 and 4 years during the Hib trial (and the pneumococcal vaccine trial in Basse) was assumed to be the same as the population aged 2 years. Age-specific rates of NTS infection during the Hib vaccine trial are unavailable, however the total number of *Salmonella* isolates is known. The incidence of NTS infection in the trial was extrapolated by multiplying the incidence of IPD by the ratio of NTS to pneumococcal isolates detected in blood.

Three studies in Basse have documented the incidence of invasive NTS and IPD (tables 1 and 3): (a) community-based surveillance of children for suspected pneumonia, meningitis or septicemia between 1989 and 1991 [6], [15], (b) surveillance during a 9-valent pneumococcal conjugate vaccine trial undertaken between 2000 and 2004 – only findings from children in the placebo group are used in this analysis [14], [16], and (c) ongoing surveillance for invasive bacterial infections which began in 2008. Methods of case ascertainment were comparable in these three studies (blood cultures were performed for all children with clinical pneumonia or symptoms or signs suggestive of sepsis or meningitis, table 1). Automated blood cultures were performed in the second and third studies and traditional microbiological methods for identification of NTS were used in all three studies.

During the 1989–91 study, the under-5 population of Upper River Region (URR) was estimated to be 24793 and approximately half the population lived in areas covered by the disease surveillance [15]. The person-years at risk in the placebo group of the pneumococcal vaccine trial were assumed to be the same as for participants assigned to vaccine. In 2008, a demographic survey showed that the under-5 population was 24480. The age range of children included in the pneumococcal vaccine trial was 6 weeks to 29 months. The estimated incidence of invasive infection among those aged 36–59 months during the vaccine trial was assumed to be half of that among those aged 24–29 months [2], [5]. Universal pneumococcal vaccination was not introduced during the observation period.

Three studies in Basse documented the prevalence of malaria parasitaemia in children aged less than 5 years during the study period (tables 1 and 3): (a) a population-based survey conducted in 1992 as part of an evaluation of insecticide treated bed nets [17], (b) surveys undertaken among children aged 15–20 months in 1994 and 23–28 months in 1995 as part of a malaria vaccine trial [18], and (c) a population-based survey undertaken in 2008 [19]. Case ascertainment was comparable in these studies, each survey being conducted at the end of the wet season with similar microscopic techniques.

**Table 3:** Data derived from Basse, Upper River Region studies.

Ref.	Location	Year	Population & (person-years) denominators	NTS isolates	Pneumococcal isolates	NTS incidence <5 years*	IPD incidence <5 years*	Prevalence malaria parasitaemia
[6], [15]	URR	1989–91	12396 (24793)	26	46	105	186	n/a
[14], [16]	U/CRR	2000–04	(16501, 0–2.5 years)†	49‡	60§	156∥	225**	n/a
††	URR	2008	24480 (24480)	7	36	29	147	n/a
[17]	URR	1992	n/a	n/a	n/a	n/a	n/a	44.7%
††	URR	1994	n/a	n/a	n/a	n/a	n/a	39%
[18]	URR	1995	n/a	n/a	n/a	n/a	n/a	35%
[19]	URR	2008	n/a	n/a	n/a	n/a	n/a	9.5%

Note: * Incidence per 100000 person-years. UUR, Upper River Region. CRR, Central River Region.

†Person-years at risk in the placebo group is calculated from Enwere et al. (2006) in which there were 330 episodes of invasive bacterial infection in vaccine and placebo groups with overall incidence of 1009 per 100000 person-years [14]; 0.5× (330/1009×10^−5^) = 16501 person-years.

‡The number of NTS isolates in the placebo group is calculated using the ratio of NTS incidence in placebo (300×10^−5^) versus vaccine (262×10^−5^) groups = 1.14 [14], and given 92 NTS isolates were detected in both groups. Thus, there were 49 NTS isolates in the placebo group (49/43 = 1.14 and 49+43 = 92).

§The number of pneumococcal isolates in the placebo group is calculated using the incidence of IPD in placebo (453×10^−5^) versus vaccine (256×10^−5^) groups = 1.77 [14], and given 94 pneumococcal bloodstream isolates were detected in both groups. Thus, there were 60 pneumococcal isolates in the placebo group (60/34 = 1.77 and 60+34 = 94).

∥NTS incidence calculated using age-specific incidence from Enwere et al., i.e. 2–5 mo: 408, 6–11 mo: 360, 12–17 mo: 334, 18–23 mo: 293, 24–29 mo: 42, 3–4 years: extrapolated as half the incidence among those 2 years of age, 42/2 = 21, <5 years: [(408/2)+(360/2)+(334/2)+(293/2)+42+21+21]/5 = 156.

**IPD incidence calculated from age-specific values [14], 2–5 mo: 363, 6–11 mo: 576, 12–17 mo: 526, 18–23 mo: 351, 24–29 mo: 339, 3–4 yrs: extrapolated as 339/2 = 170. Calculation of <5 years incidence of IPD takes into account that 71% of pneumococcal isolates were derived from blood [14]: 0.71[(363/2)+(576/2)+(526/2)+(351/2)+339+170+170]/5 = 225.

††authors' unpublished data.

We compared temporal patterns of NTS infection with indices of malaria infection at the closest time point for which malaria data were available. To provide a prevalence measure of NTS infection relative to other pathogens, and for partial control of confounding due to variable blood culture practices and age, we calculated the ratio of NTS to pneumococcal (P) isolates detected in blood cultures in all the studies (NTS∶P). We also compared trends in the incidence of blood culture positive IPD with the incidence of NTS infection in children less than 5 years of age in all the studies.

At the end of 2004, the government guideline for first-line therapy of mild malaria was changed from chloroquine to a combination of chloroquine, sulfadoxine and pyrimethamine. Artemether plus lumefantrine was introduced through government health facilities in 2008. Quinine was the treatment of choice for severe malaria throughout the observation period. Multiple indicator surveys in 2000 and 2006 documented the proportion of children under 5 years of age sleeping under insecticide treated nets as 14.5% and 49% respectively [20], [21].

Data were extracted directly from publications and derived from current studies. Incidence rates were calculated assuming Poisson distributions.

All the clinical trials from which data presented in this paper have been derived were approved by the Gambia Government/MRC Joint Ethics Committee and all research study participants provided written informed consent.

## Results

### Fajara

**Figure 1:**
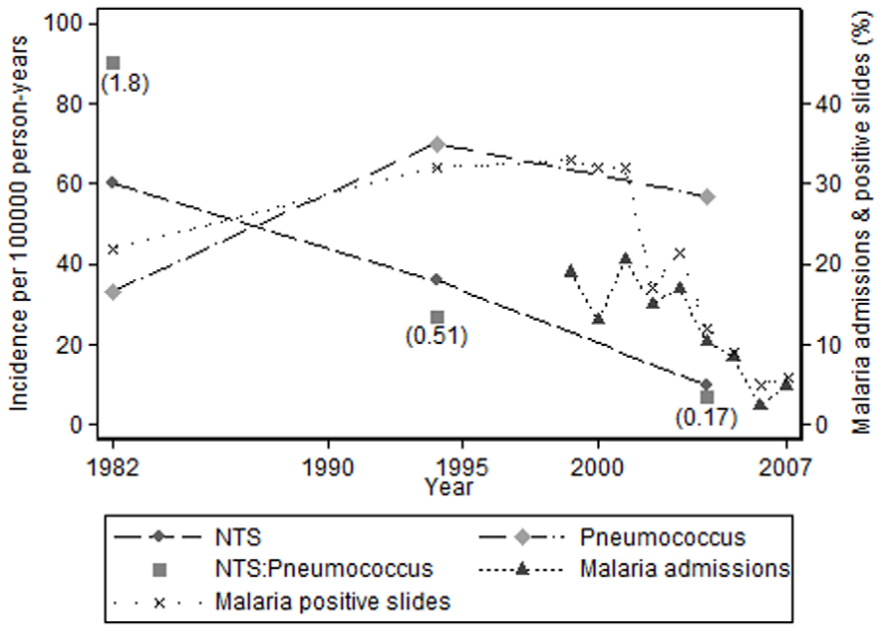
Fajara: Temporal trends of non-typhoid salmonella and pneumococcal bacteraemia, and malaria indices.

Over a 69 month period from 1979 until 1984, pathogenic bacteria were isolated from 259 patients less than 5 years of age (approximately 20000 population and 115000 person-years at risk) who presented to the MRC Hospital, Fajara; 69 were NTS and 38 were pneumococci (NTS∶P = 1.8) [4] (Table 2). During 32 months of a Hib conjugate vaccine trial (1993–95), 2408 blood cultures were taken and 39 *Salmonella spp*. and 76 pneumococci were isolated from blood (NTS∶P = 0.51) [12]. During 16 months from 2003 until 2005, 871 blood cultures were taken from children under 5 years (approximately 38322 population and 51096 person-years at risk) who presented at the MRC Hospital; 93 pathogens were isolated, 5 NTS and 29 pneumococci (NTS∶P = 0.17) [13]. Over the study period, the incidence of NTS infection among those less than 5 years fell by 84% (95% CI: 60, 95), from approximately 60 to 10 cases per 100000 person-years (figure 1). The estimated incidence of IPD did not fall over this time, with point estimates of 33, 70 and 57 cases per 100000 person-years in the three studies (table 2 & figure 1).

The fall in invasive NTS infection followed a similar pattern to the fall in malaria infection (table 2 & figure 1). The proportion of admissions attributed to malaria fell over a period of 6 years, from 20.5% in 2001 to 5% in 2007 [11]. The percentage of positive malaria slides in outpatients was stable between 1979 and 2001, at around 20% to 30% (authors' unpublished data [BG]); this then fell over a period of 9 years, from 33% in 1999 to 6% in 2007 (figure 1).

### Basse

**Figure 2:**
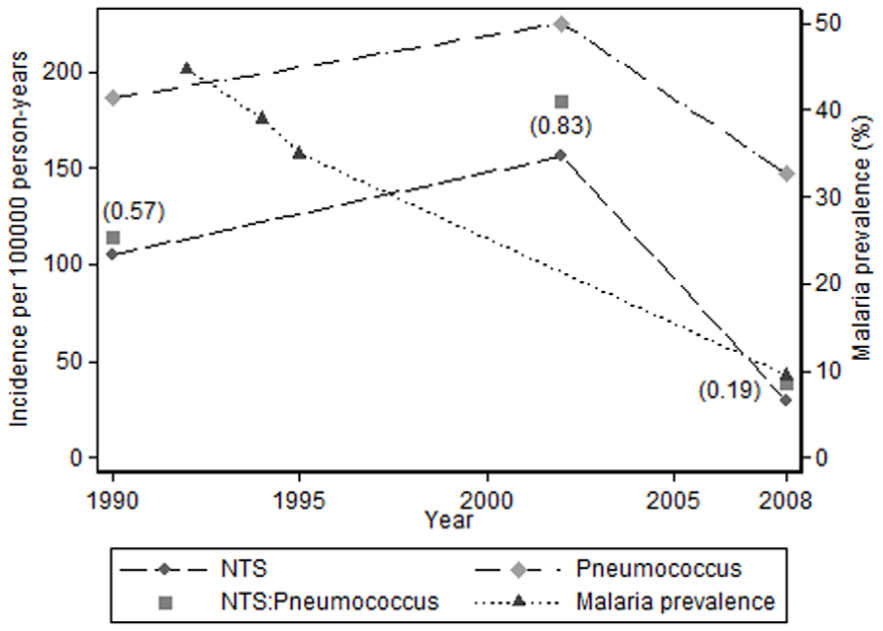
Basse: Temporal trends of non-typhoid salmonella and pneumococcal bacteraemia, and malaria prevalence.

Twenty-four months of community-based surveillance for invasive bacterial infection under 5 years of age was conducted between 1989 and 1991 (24793 person-years at risk) [15] (Table 3). Twenty-six NTS and 46 pneumococci were isolated (NTS∶P = 0.57, figure 2). From 2000 until 2004, during a pneumococcal conjugate vaccine trial, 49 NTS and 59 pneumococci were isolated from blood in separate episodes in the placebo group (NTS∶P = 0.83) [14], [16]. During 12 months of surveillance in 2008 (24480 person-years at risk), 7 NTS and 36 pneumococci were isolated from children under 5 years of age (NTS∶P = 0.19) (authors' unpublished data [GM]). The incidence of invasive NTS among those less than 5 years fell by 73% (95% CI: 37, 88) over the period of the three studies, from 105 to 29 cases per 100000 person-years (figure 2). The incidence of IPD in non-vaccinated children remained stable with point estimates of 186, 225 and 147 cases per 100000 person-years respectively (figure 2).

The end of transmission season prevalence of malaria parasites in cross-sectional surveys of young children resident in URR fell from 45% (1992), 39% (1994) and 35% (1995) to 10% in 2008 (figure 2) [17]–[19].

## Discussion

As far as we are aware, this is the first report to document reduced rates of invasive NTS infection in children associated with reductions in malaria.

The time point at which the incidence of NTS infection in Fajara began to fall is uncertain, but it is somewhere between 1994 and 2004 (figure 1). Malaria infection in Fajara began to fall around 2001, and markedly so in 2003 [11]. Thus, the decline in the incidence of NTS followed a comparable time course to the fall in malaria. In Basse, the incidence of NTS infection fell between 2002 and 2008 (figure 2), showing a similar temporal pattern to the recent decline of malaria in The Gambia [11].

This study has several weaknesses inherent in a retrospective analysis of data collected for other purposes. Temporal changes in disease would have been best described by continuous data collection. At Basse, blood cultures were collected from children identified in populations under surveillance whilst at Fajara samples were obtained from children who came from a wide area and chose the MRC hospital as their centre for care. Thus, the Basse data are more representative of the population than those of Fajara, but the similar patterns of falling malaria and NTS infection in both locations strengthen the case for an association between malaria and invasive NTS infection. We made intuitive assumptions on the catchment populations for the MRC Hospital at Fajara, and so these incidence estimates are only approximate. Calculation of disease incidence assumed similar under-5 age structure in the Hib and pneumococcal vaccine trials as in the other studies providing data. Likewise, the association of NTS and IPD with age strata less than 5 years was assumed to be the same for both organisms. If these assumptions are false, the interpretation of the data would not change but be strengthened as the true difference between the incidence of IPD and NTS in the latter studies would be greater than that which was observed.

There were differences in selection criteria for blood culture in the Fajara studies, where blood cultures may have been collected more assiduously during the vaccine trial than during routine hospital admissions. However, the comparability between NTS studies conducted in Basse should be good as similar criteria for blood cultures were used in the three studies. To reduce confounding by variable patient selection or factors reducing the risk of any bacteraemia, we calculated rates of pneumococcal blood stream infections during the same surveillance periods. Greatly reduced NTS∶P ratios of blood culture isolates suggests that there has been a marked decline in the prevalence of NTS infection which is not due to changes in selection criteria for blood culture nor due to non-species specific reductions in rates of childhood bacteraemia. Although automated blood culture systems were introduced during the study period this is unlikely to have changed the sensitivity of NTS detection. Methods for calculation of malaria admissions and slide positivity in Fajara were consistent throughout as were the population-based malaria prevalence survey techniques used in Basse. Unfortunately, we do not have detailed information on the age distribution of children under the age of 5 years admitted to the hospital in Fajara and changes in this pattern could have influenced the apparent decline in the prevalence of malaria. However, experience of those involved in patient care at the MRC hospital indicates that if there has been a change in age pattern this has been small. As malaria prevalence estimates from Basse were derived from population-based surveys less than 5 years of age, these data are not subject to bias due to changes in age. The similar pattern and magnitude of NTS reduction in parallel with reduced malaria prevalence in Fajara and Basse suggests that the age-related bias in the Fajara data is not substantial. Therefore, our interpretation of a potential association between reduced prevalence of childhood malaria and reduced incidence of NTS infection remains valid.

A decline in the incidence of NTS infection in parallel with a decline in malaria does not prove cause and effect and we have considered other possible explanations for our findings. Improved socio-economic and environmental conditions may be associated with reduced risk of childhood malaria and enteric infections associated with fecal-oral transmission, and hence reduced rates of NTS infection. One study has documented reduced rates of diarrhoea in The Gambia between 1979 and 1993 [22]. This study however, predates the observed reductions in malaria (from 2001–2007 [11]) and NTS infection (from 1994–2004 in Fajara and 2002–2008 in Basse [figures 1 and 2]). Furthermore, gastrointestinal infection remains prevalent in URR with 25% of carers in a 2008 survey reporting recent diarrhea among children less than 5 years of age (authors' unpublished data [DS]). Rainfall may relate to both the intensity of malaria transmission and risk of NTS infection. The annual rainfall in the Greater Banjul area has fluctuated between approximately 1100 millimeters in 1999 and 850 millimeters in 2007 [11]. Annual rainfall in Basse from 1998 until 2008 remained stable at between 700 millimeters and 1000 millimeters. Use of trimethoprim-sulphamethoxazole for community-based pneumonia management may have influenced rates of NTS and malaria infection although resistance of NTS to this drug is significant in Fajara [13] and Basse. Likewise, sulfadoxine has antibacterial activity and it was widely used in combination therapy for uncomplicated malaria and combination sulfadoxine and pyrimethamine remains the mainstay of malaria intermittent preventive therapy. HIV and malnutrition are risk factors for NTS infection. HIV prevalence has remained less than 2% in The Gambia during the past 20 years, and Multiple Indicator Cluster Surveys in 1996, 2000 and 2005/6 do not suggest substantial changes in patterns of malnutrition [20], [21], [23]. Although some of the competing explanations for our findings may have influenced NTS and malaria infection to some degree, it seems the dramatic fall in incidence of malaria in the Gambia is the most likely reason why the incidence of NTS infection has fallen so markedly.

Previous epidemiological, clinical and pathophysiological studies support our hypothesis that the reduction in NTS infection which has been observed in The Gambia is likely to be associated with the substantial fall in malaria which has been recently observed in the country. Compared to other forms of bacteraemia, invasive NTS has previously been associated with anaemia [4], [5], [7]–[10], splenomegaly [5], [7]–[9], recent [5] and concurrent malaria parasitaemia [4], [7]–[10]. The link between NTS and anaemia suggests a possible mechanism for the observed association between the decline in the incidence of NTS and the decline in the incidence of malaria in The Gambia as a reduced rate of anaemia has been observed in The Gambia in association with the reduction in malaria [11]. A further epidemiological factor which may contribute to reduced NTS infection is the increase in median age of children with malaria in The Gambia [11] as infants and young children are at the greatest risk of NTS infection. Possibly the strongest epidemiological evidence linking reductions of NTS and malaria are the elegant descriptions from The Gambia [4], [6] and Kenya [5] illustrating the intimate relationship between seasonal changes in the incidence of malaria and NTS infection while the prevalence of NTS in stool was relatively stable throughout the year [4], [5]. A number of ways in which malaria might reduce host defenses against invasive NTS infection have been suggested [1]. These include: altered humoral immunity and antibody response to *Salmonella* antigens, increased circulating immune complexes which inhibit Fc receptor-mediated phagocytosis, reduced complement levels, alteration of phagocytic and bactericidal function by ingested malaria pigment, altered splenic function, free haemoglobin resulting in production of methaemalbumin and increased availability of iron stimulating bacterial growth through the many iron uptake pathways possessed by NTS.

An intervention study in The Gambia has shown that the use of insecticide treated bed nets was associated with reductions in overall child mortality of a magnitude greater than what was expected due to reductions in malaria-attributable child mortality alone [24]. Furthermore, there has recently been a dramatic decline in child mortality in The Gambia which has paralleled the decline in the incidence of malaria (personal communication, M Jasseh, 14 December 2009). This reduction is far greater than would have been anticipated on the basis of the proportion of deaths previously attributed directly to malaria. Similar changes have been observed on the coast of Kenya, where the incidence of NTS infections has also declined in parallel with marked reductions in malaria transmission (personal communication, K Marsh, 2 November 2009). One possible explanation for the greater than expected impact of effective malaria control on overall mortality in children living in malaria endemic areas is the prevention of invasive infections by some bacterial species, as suggested in the current study.

If similar findings to ours are found in other locations which have experienced reductions in the incidence of malaria, the implications are substantial. Investments in research and public health to control malaria may have added value, reducing rates of NTS infection and the associated child mortality, in addition to reducing mortality related directly to malaria.
